# Increased Health and Wellbeing in Preschools (DAGIS) Study—Differences in Children’s Energy Balance-Related Behaviors (EBRBs) and in Long-Term Stress by Parental Educational Level

**DOI:** 10.3390/ijerph15102313

**Published:** 2018-10-21

**Authors:** Elviira Lehto, Carola Ray, Henna Vepsäläinen, Liisa Korkalo, Reetta Lehto, Riikka Kaukonen, Eira Suhonen, Mari Nislin, Kaija Nissinen, Essi Skaffari, Leena Koivusilta, Nina Sajaniemi, Maijaliisa Erkkola, Eva Roos

**Affiliations:** 1 Folkhälsan Research Center, Topeliuksenkatu 20, 00250 Helsinki, Finland; carola.ray@folkhalsan.fi (C.R.); reetta.lehto@folkhalsan.fi (R.L.); riikka.kaukonen@folkhalsan.fi (R.K.); eva.roos@folkhalsan.fi (E.R.); 2 Faculty of Educational Sciences, University of Helsinki, P.O. Box 9, 00100 Helsinki, Finland; eira.suhonen@helsinki.fi (E.S.); nina.sajaniemi@helsinki.fi (N.S.); 3 Department of Food and Nutrition, University of Helsinki, P.O. Box 66, 00100 Helsinki, Finland; henna.vepsalainen@helsinki.fi (H.V.); liisa.korkalo@helsinki.fi (L.K.); essi.skaffari@helsinki.fi (E.S.); maijaliisa.erkkola@helsinki.fi (M.E.); 4 Department of Early Childhood Education, The Education University of Hong Kong, 10 Lo Ping Road, New Territories, Hong Kong; manislin@eduhk.hk; 5 School of Food and Agriculture, Seinäjoki University of Applied Sciences, P.O. Box 412, 60320 Seinäjoki, Finland; kaija.nissinen@seamk.fi; 6 Department of Social Research, Faculty of Social Sciences, University of Turku, Assistentinkatu 7, 20500 Turku, Finland; leeko@utu.fi

**Keywords:** energy balance-related behaviors, preschool, children, socioeconomic differences, needs assessment, long-term stress, cortisol, screen time, sugary foods and beverages, fruit and vegetables

## Abstract

This paper describes the Increased Health and Wellbeing in Preschools (DAGIS) survey process and socioeconomic status (SES) differences in children’s energy balance-related behaviors (EBRBs), meaning physical activity, sedentary and dietary behaviors, and long-term stress that serve as the basis for the intervention development. A cross-sectional survey was conducted during 2015–2016 in 66 Finnish preschools in eight municipalities involving 864 children (3–6 years old). Parents, preschool personnel, and principals assessed environmental factors at home and preschool with questionnaires. Measurement of children’s EBRBs involved three-day food records, food frequency questionnaires (FFQ), seven-day accelerometer data, and seven-day sedentary behavior diaries. Children’s long-term stress was measured by hair cortisol concentration. Parental educational level (PEL) served as an indicator of SES. Children with low PEL had more screen time, more frequent consumption of sugary beverages and lower consumption of vegetables, fruit, and berries (VFB) than those with high PEL. Children with middle PEL had a higher risk of consuming sugary everyday foods than children with high PEL. No PEL differences were found in children’s physical activity, sedentary time, or long-term stress. The DAGIS intervention, aiming to diminish SES differences in preschool children’s EBRBs, needs to have a special focus on screen time and consumption of sugary foods and beverages, and VFB.

## 1. Introduction

Children’s energy balance-related behaviors (EBRBs), such as physical activity (PA), sedentary behavior, and food consumption, are important in determining children’s current and later health, including being overweight and obese [1,2]. Socioeconomic status (SES)-related differences in some EBRBs already seem prevalent among young children in Finland [3,4]; however, research conducted among preschool-aged children is scarce. Stress, measured both as imbalanced short-term stress regulation and long-term stress, is a direct predictor of obesity in children alongside EBRBs; however, stress may also predict obesity through unhealthy EBRBs [5]. Imbalanced stress regulation is associated, for example, with unhealthy eating behavior among preschool children [6]. Long-term stress, similar to unhealthy EBRBs, is often more prevalent among children from low SES families [7].

EBRBs established in childhood track into adulthood [8]. Consequently, interventions aiming to decrease SES differences in both EBRBs and long-term stress should be conducted during the early years to prevent inequalities in health later in life. Acknowledging the growing number of obesity-prevention interventions among preschoolers, many of the interventions have not strongly impacted targeted EBRBs [9,10]. Improved rates of effectiveness appear in those multi-component interventions conducted in a structured setting such as preschool, combined with a strong family engagement [9,10]. Moreover, socioecological models suggest that EBRBs are influenced by factors at multiple levels: intrapersonal, such as age; environmental setting, such as preschool and family; and societal level, such as SES [11]. Hence, research focusing on several levels and settings affecting preschoolers can more widely identify new modifiable factors to use in interventions promoting EBRBs and balanced stress regulation.

A new project, the Increased Health and Wellbeing in Preschools (DAGIS) study [12], firstly aimed to determine the existence of SES differences in children’s EBRBs and long-term stress. Secondly, the project aimed to find innovative and effective methods to tackle these SES differences. Thirdly, the DAGIS study aimed to develop and conduct an intervention to improve children’s EBRBs and stress regulation, simultaneously diminishing SES differences in EBRBs. The intervention mapping (IM) framework stresses a systematic planning of health-promotion programs [13] and emphasizes the importance of the first step in IM, called needs assessment. The needs-assessment phase involves creating a logic model of the health problem, based on existing knowledge. The model depicts factors influencing the health problem at different ecological levels and also illustrates the assumed causal links between these factors. Since Finnish children with lower SES have been found to be overweight or obese more often [14], the need to examine SES differences in the predictors of overweight, such as EBRBs and long-term stress, was apparent. Due to limited data and number of studies concerning preschoolers in the Finnish context, the DAGIS study’s first phase involved a comprehensive needs assessment. The needs assessment aimed to recognize SES differences in preschoolers’ EBRBs and long-term stress. The assessment included a review of existing literature (not published) and focus group interviews with preschool personnel and parents [15,16,17].

In addition to the above-mentioned issues regarding needs assessment, the DAGIS study included an extensive survey [12]. As preschool-aged children spend most of their time at home and preschool, the survey concentrated equally on both settings. This paper describes the survey-participant recruitment; the comprehensive data-collection methods at different levels in preschool and home settings; and, in more detail, the data collection of the outcome variables: EBRBs and long-term stress. This paper also presents parental educational level (PEL) differences in both preschool children’s EBRBs and long-term stress, which serve as grounds for subsequent intervention. In EBRBs, we concentrate specifically on physical activity, sedentary behaviors, screen time, and the consumption of sugary foods and beverages, as well as the consumption of vegetables, fruit, and berries (VFB).

## 2. Materials and Methods

### 2.1. Study Design

This survey is part of the DAGIS study that aimed to diminish socioeconomic differences in preschool children’s EBRBs [18]. The design of, and rationale for, the DAGIS survey have been described in detail elsewhere [12]. The first phase of DAGIS constituted a cross-sectional survey, conducted between autumn 2015 and spring 2016. The survey aimed to examine EBRBs and long-term stress among three- to six-year-old preschoolers, to recognize SES differences, and to identify the factors associated with EBRBs and long-term stress at home and at preschool. The study was approved by the University of Helsinki Ethical Review Board in the Humanities and Social and Behavioral Sciences in February 2015 (#6/2015).

**Figure 1:**
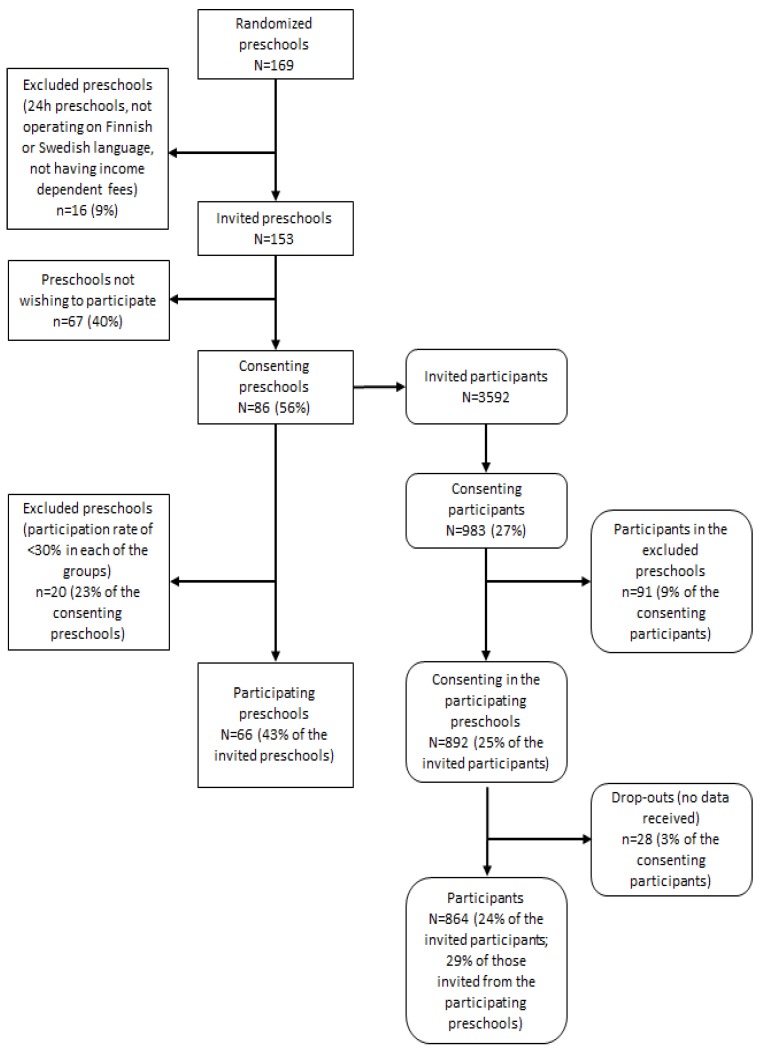
Participation and exclusion of preschools and families in the DAGIS survey.

Municipalities situated in Southern and Western Finland were selected according to their location, that is, being a convenient distance from the research centers, and their socioeconomically diverse populations. To ensure municipalities had socioeconomically diverse populations, our selection stressed the following indicators: the Gini coefficient of the municipality and the proportion of single parents and people with low educational level in the municipality, which are presented in the national statistics [19]. Of the municipalities invited to the study, eight of the eleven (Porvoo, Loviisa, Vantaa, Hyvinkää, Lohja, Seinäjoki, Kauhajoki, and Kurikka) agreed to participate (participation rate 73%). Participating municipalities gave a list of all municipal preschools and private preschools from which the municipalities purchase early education services. Preschool eligibility criteria for the study involved: (1) having at least one group consisting of 3–6-year-old children; (2) providing early education only during the daytime; (3) being Finnish or Swedish speaking (official languages of Finland); and (4) charging income-dependent fees (all municipal preschools have reduced fees for low-income families). We randomized all preschools listed by the municipalities with a list randomization program (https://www.random.org/lists/) and contacted 169 of them in the randomized order. We excluded 16 preschools based on the eligibility criteria and thus, invited 153 preschools to participate in the study. Figure 1 presents the recruitment, exclusion and drop-out rates on preschool and family levels. Consenting preschools numbered 86 (56% of the invited).

All families with a preschool child aged three to six years were invited to the study by an informational letter with attached consent sheet inserted in the children’s preschool lockers. We aimed to collect data on 800 children, based on power calculations [12]. Parents gave their written consent for a total of 983 children. We excluded, however, preschools with a total parental consent rate of less than 30% in all of the groups. This exclusion saved research resources by removing the need to travel and conduct surveys in preschools with a low number of participants. Preschools excluded due to a too-low consent rates accounted for 20 preschools, involving 91 children. The final number of participating preschools was 66 (43% of invited) and the number of children with parental consent was 892 (25% of invited). Of all consenting families, we have at least some data (based on a sample, questionnaire or accelerometer) on 864 children, which we consider the final number of participating children in the study (Figure 1).

We also invited the principals and personnel of the preschools to participate in the study by answering separate questionnaires (Table A1). Each preschool personnel member received a questionnaire, and each preschool group had one contact person who filled in an additional questionnaire. In the participating preschools, 60 principals (91% of the invited) and 379 members of the personnel (79%) answered the questionnaire.

The DAGIS study uses the socioecological model [11] as a framework for factors influencing children’s EBRBs and stress regulation. We wanted data in the DAGIS survey to cover EBRBs and stress regulation on several levels. The first level comprises children’s individual level; the second level includes physical and social environment both at home and at preschool level; and at the outermost level incorporates parental SES and neighborhood SES of preschools. Data collection involved several kinds of measurements: biological measures, accelerometer data, observations, and questionnaires. We briefly describe the data collected in the home and preschool setting and on SES, but give more detail on the data measurements of children’s EBRBs and long-term stress and of PEL. Table A1 in the Appendix A lists the various data-assessment methods at different levels, what they measure, and the number of observations available in the data.

### 2.2. Data Collection Procedure

The data collection took place in 66 preschools between September 2015 and April 2016. The period of data collection in each preschool was one week (seven days). The data collection week started on Day 1 between Monday and Thursday, when research staff (two to four persons, depending on group and participant number) visited a preschool for half a day. During the first day, research staff measured the height, weight, and waist circumference of participating children, and set accelerometers on the right side of participating children’s hips (see the description of the variables for details). To gain a more objective view of the topic and to lower the burden of the preschool personnel, the research staff observed and filled in a questionnaire on both indoor and outdoor facilities and the lunch situation of the preschool groups. Research staff gave preschool personnel both study materials to distribute among parents (guardians’ questionnaire, temperament questionnaire, saliva sample materials, and accelerometer-related diary), and study questionnaires to distribute among food-service staff. Finally, research staff described to the preschool personnel what they were expected to do during the study week and showed them how to carry out the required tasks. These tasks included filling in children’s food records during preschool hours, collecting children’s hair and saliva samples, and filling in personnel questionnaires. The same information which was provided orally, was also given to the preschool personnel in writing. The principals filled in a web-based questionnaire.

Parents had received the food frequency questionnaires (FFQ) and food records by mail approximately one week before the study week and returned them to the preschool together with other questionnaires and samples. Research staff (two persons) collected all the study materials after the seven days and interviewed the food-service staff about foods served at preschool. After the data collection, we reminded the principals, the contact person of the preschool groups, and parents two to three times if some questionnaires or samples were missing.

### 2.3. Measurements of Major Outcomes, Parental Educational Level, and Covariates

#### 2.3.1. Physical Activity and Sedentary Time

Objective moderate-to-vigorous physical activity (MVPA) and sedentary time were measured with Actigraph wGT3X-BT accelerometers (Actigraph, LLC, Pensacola, FL, USA). Children wore accelerometers for seven days, 24 h per day. Parents marked in a diary the hours children had spent at preschool as well as possible non-wearing hours of the accelerometers due to water-based activities, for example. We used a 15-s epoch length when downloading data from the accelerometers and set the non-wearing time at 10 min or more consecutive zeros [20]. To form the sedentary time variable, we used cut points of 0–100 counts per minute recognized by Evenson et al. [21], as these reportedly classify sedentary time accurately among 5–15-year-old children [22]. For the MVPA, the set cut point was at least 2269 counts per minute [21]. Criteria to form variables indicating sedentary time and MVPA required having available data for at least four days, from which at least one day was a weekend day, with a minimum of 600 min per day. Sedentary time was divided by the time the child was wearing the accelerometer and multiplied by 60 min, to calculate the average sedentary time per hour. We excluded data from days parents had reported their child being sick or absent from preschool. We also excluded the night sleeping hours but not the possible daytime nap times.

#### 2.3.2. Screen Time

Screen time and other types of sedentary behaviors were reported by parents, who filled in a seven-day sedentary behavior diary for the same days that the children wore the accelerometers. This sedentary behavior diary is a translated and modified version of a previously validated diary [23]. Parents assessed the frequency and time in hours and minutes that their child spent in front of a television, DVD/video, computer or tablet/smartphone. Daily screen time is a composition variable of all the above-mentioned types of screen time.

#### 2.3.3. Food Consumption

Measuring children’s food consumption involved both FFQ and food records. We designed a 47-item FFQ to assess children’s dietary quality in general, paying specific attention to capturing the consumption patterns of vegetables, fruit, and berries (fresh vegetables; cooked and canned vegetables; fresh fruit; and fresh and frozen berries), sugary everyday foods (flavored yogurt and quark; puddings; sugar-sweetened cereals and muesli; berry, fruit and chocolate porridge with added sugar; and berry and fruit soups with added sugar), sugary treats (ice cream; chocolate; sweets; cakes, cupcakes, sweet rolls, Danish pastries, pies and other sweet pastries; and sweet biscuits and cereal bars), and sugary beverages (soft drinks; flavored and sweetened milk- and plant-based drinks; and sugar-sweetened juice drinks). The FFQ was based on previous studies among Finnish children [3,24]. The respondent (parent or legal guardian) reported in the FFQ how many times during the past week the child had consumed different foods outside preschool hours. The FFQ included three answer columns, namely “not at all”, “times per week” and “times per day”, with instructions to either tick the “not at all” box or to write a number in one of the other columns. Instructions to parents involved completing the FFQ initially; and later, the three-day food record on pre-set dates.

Parents filled in a three-day food record, including information of two weekdays and one weekend day. These three days were not always consecutive. Preschool personnel filled in all foods eaten during preschool hours on a separate pre-coded food record on the two weekdays matching the home food record. Development and usage of a validated Children’s Food Picture Book supported portion-size estimation [25]. FFQs and food records were checked by trained research assistants and follow-up phone calls were made as necessary to complete missing details of foods consumed. Research assistants entered the food record data using AivoDiet dietary software, which employs the national food composition database Fineli [26]. Municipal food services provided the recipes for the food served at preschools in five out of eight municipalities. When necessary, new food items were added to the database and database recipes were modified, or new ones created, according to parents’ reports. Construction of variables measuring the consumption of sugary everyday foods, sugary treats, sugary beverages, and VFB allowed inclusion of, as far as possible, the same foods as listed above regarding FFQ. The minor differences between the variables are as follows: the food record-based variable defining sugary everyday foods does not include berry, fruit and chocolate porridges with added sugar but includes milk shakes and vanilla sauce; variable defining sugary treats lacks berry and fruit pies, and variable defining vegetables includes only fresh vegetables and vegetables served as side dish, whereas, in the FFQ, the participants were instructed to report also vegetables eaten as part of the dishes.

#### 2.3.4. Long-Term Stress

We measured children’s long-term stress with hair cortisol concentrations (HCC), which capture long-term integrated cortisol levels [27]. Trained preschool personnel collected hair samples from the posterior vertex of the scalp of the children. A hair lock of approximately 40 hairs was tied together and cut as close to the scalp as possible. If the hair was too short to be tied together, no hair sample was taken. The scalp end of the hair sample was marked, the sample packed in foil and put in a small plastic bag to send to a laboratory for analysis. In the laboratory, the strands were lined up and cut into two separate 2-cm segments. The laboratory followed the protocol of Davenport et al. [28] for the washing of hair and steroid extraction. Cortisol determination involved commercially available immunoassay with chemiluminescence detection (CLIA, IBL-Hamburg, Germany). The intra assay and inter assay coefficient of variance of this assay is below 8%. We report the mean HCC (pg/mg) of the two segments, which indicates HCC of approximately the past four months.

#### 2.3.5. Parental Educational Level

The survey questionnaire assesses different parental factors related to a child’s physical activity, sedentary time, screen time, and eating behaviors. The educational level of the parent who filled in the survey questionnaire serves as an indicator of parental educational level (PEL). The parent who gave consent had reported the highest educational level for both themselves and their partner living in the same household, if relevant. The response categories for the question “What is your highest educational achievement?” were: (1) comprehensive school; (2) vocational school; (3) high school; (4) bachelor’s degree or college; (5) master’s degree; and (6) licentiate/doctor. To assure a sufficient size of the educational level groups, the answers were categorized as: (1) low educational level (including 1–3); (2) middle educational level (including 4); and (3) high educational level (including 5–6). In the survey questionnaire, the responding parent indicated being the child’s mother, stepmother, father, stepfather or other guardian. None of the respondents was a stepmother or stepfather. Other guardians (*n* = 4) were excluded from the analyses. Combining the educational level information from the consent form with the parental status from the survey questionnaire allowed forming a variable indicating PEL. Not all questionnaires, namely survey questionnaire and FFQ, were filled in by the same parent and, moreover, no information exists regarding who completed the sedentary behavior diary and food record. Therefore, we decided to use PEL of the guardian who filled in the main survey questionnaire.

#### 2.3.6. Covariates

We adjusted the analyses for children’s gender (girl/boy), age (continuous variable calculated by subtracting the birthdate from the research date), and the respondent’s parental status (mother/father), according to the parental report. Additionally, we adjusted the analysis according to the season of conducting the research (September–October, November–December, or January–April). The FFQ measures food consumption frequency outside preschool hours; consequently, we controlled analyses concerning consumption frequency of sugary everyday foods, treats, and beverages or VFB also for the time spent in preschool (hours per week), which was reported by the parents.

### 2.4. Statistical Methods

Presented are sample characteristics of the untransformed variables as percentages or means, standard deviations, and range. Examination of PEL differences in the EBRBs used general linear models, adjusting for covariates. Prior to these analyses, we excluded outliers in the distance of three standard deviations or more from the mean (sedentary time *n* = 1, MVPA *n* = 5, screen time *n* = 4, consumption frequency of all sugary everyday foods, treats, and beverages counted together *n* = 13, consumption frequency of sugary everyday foods *n* = 13, consumption frequency of sugary treats *n* = 18, consumption frequency of sugary beverages *n* = 18, consumption frequency of VFB *n* = 11, consumption frequency of V *n* = 8, and consumption frequency of FB *n* = 14). In comparing multiple groups, we used the LSD test with Sidak corrections. The variables derived from the FFQ, which measured consumption frequency of sugary everyday foods, sugary treats, sugary beverages, and VFB, were positively skewed; therefore, we used logarithmically transformed variables in the analyses. Regarding food record, the variables measuring the consumption of sugary everyday foods, sugary treats, sugary beverages, and VFB, and also the HCC variable were positively skewed; however, a logarithm transformation was insufficient to correct the skewness of the distribution. Therefore, we categorized the variables measuring the consumption of sugary everyday foods, sugary treats, sugary beverages, and VFB and HCC into quintiles and compared the odds of being in the highest quintile with the lower quintiles using logistic regression analyses. In the logistic regression analyses, the highest PEL group served as the reference group. The level of statistical significance was set at *p* < 0.05. We analyzed the data with IBM SPSS version 25.0 (IBM, Armonk, NY, USA).

## 3. Results

Table 1 describes the study participants. Approximately half of the participating children were girls and the children’s mean age was 4.7 (SD 0.9) years. Guardians who filled in the questionnaires were mainly mothers: 88% regarding the survey questionnaire and 93% regarding the FFQ. Approximately 29% of the guardians had high school level education or less, 40% had a bachelor’s degree or equivalent, and 29% had at least a master’s degree.

Table 2 presents the PEL differences in children’s EBRBs examined with a general linear model. Children with low PEL had higher screen time than children with higher PEL (*p* < 0.01) (F(2,713) = 5.01, *p* = 0.01). No significant differences existed in children’s sedentary time (F(2,722) = 0.56, *p* = 0.57) or children’s MVPA (F(2,718) = 0.23, *p* = 0.79) according to PEL.

Table 2 also presents results concerning food consumption measured with FFQ. No PEL differences occurred in the total frequency of the consumption of sugary foods, treats, and beverages, that is, the sum of all variables (F(2,700) = 2.78, *p* = 0.06). When separately examining different groups of sugary foods and beverages, a PEL difference existed in the consumption frequency of sugary beverages (F(2,704) = 3.43, *p* = 0.03). This difference indicates children with low PEL had a higher consumption frequency than children with high PEL (*p* = 0.02). The consumption frequency of sugary everyday foods revealed PEL differences (F(2,707) = 3.03, *p* = 0.05). In pairwise comparisons of the PEL groups, however, no differences were apparent. The consumption frequency of sugary treats did not reveal PEL differences (F(2,703) = 0.38, *p* = 0.68). According to the FFQ, PEL differences neither occurred in the daily consumption frequency of vegetables, fruit, and berries examined together (F(2,709) = 0.69, *p* = 0.50) nor in separate examination of the consumption frequency of vegetables (F(2,715) = 2.42, *p* = 0.09) and fruit and berries (F(2,712) = 0.11, *p* = 0.90).

Table 3 illustrates PEL differences in children’s consumption of sugary everyday foods, sugary treats, sugary beverages, vegetables, and fruit and berries, as well as children’s long-term stress examined with logistic regression analyses. Children with middle PEL were at higher risk of being in the highest quintile of the consumption of sugary everyday foods (g/MJ/day) than children with high PEL. Children with low PEL had a higher risk of consuming less vegetables than children with high PEL. No PEL differences were found in the consumption of sugary treats, sugary beverages, or fruit and berries.

Table 3 shows no PEL differences were found in the HCC.

## 4. Discussion

This paper describes the comprehensive DAGIS survey examining EBRBs and long-term stress among preschool children, which serves as the basis of an intervention. The present study examined PEL differences in children’s EBRBs and long-term stress. Children with low PEL had higher screen time and higher consumption frequency of sugary beverages than children with high PEL. Children with low PEL, compared to children with high PEL, were also less likely to consume vegetables, when consumption was defined as grams and adjusted for daily energy intake. Children with middle PEL were at higher risk of consuming more sugary everyday foods, than children with high PEL. No PEL differences existed in children’s sedentary time, MVPA, or long-term stress levels.

When comparing our results to other recent studies that have examined preschool-aged children’s PA with accelerometers, the amount of MVPA in this study seems to be somewhat lower [30,31]. Similar to our results concerning children’s PA, little evidence exists regarding SES differences among preschoolers [32]. For example, a recent Finnish study among 6–8-year-olds [4] showed a lack of SES differences in total PA, measured by questionnaires filled in by parents together with their child. Kaikkonen et al. [14], however, earlier demonstrated how Finnish preschoolers and first-grade schoolchildren meet PA recommendations slightly more often when their mothers had higher than lower education. These results were, however, based on questionnaire data [14]. Overall, a systematic review on correlates of children’s and adolescents’ PA stated that SES differences in PA level do not yet show in young children, but emerge only later in childhood [33].

Stronger associations generally occur between SES and screen time [32]. Similarly, our study associated higher screen time more with low-PEL than high-PEL children. Previously, associations between either higher PEL or income and lower screen-based sedentary behavior occurred only among Finnish boys [4]. Most previous studies have conceptualized screen time as television viewing and computer and video game playing [32]. Since tablets and smartphones have become increasingly popular electronic devices, we considered including them in our study important to avoid underestimating children’s screen time. A recent study that also included hand-held devices in their description of screen time, reported somewhat higher levels of screen time among Australian 2–5-year-olds, compared to our results [34].

Healthier eating, defined as high consumption of fruit and vegetables and low consumption of sugary foods or beverages, has been previously associated with high PEL among preschool-aged children in several countries [35,36]. An Australian study [36] revealed PEL differences in the likelihood of having food habits closer to dietary guidelines already before preschool age, and continuously throughout the four-year research period. A Finnish study [37], however, revealed maternal educational level differences in the healthy-eating index among six-year-olds, whereas paternal educational level differences were prevalent already among three-year-olds. Among younger children, however, no PEL differences were observed [37]. Another Finnish study among 6–8-year-olds did not find any PEL or income level differences in children’s consumption of VFB or sugary foods and beverages [38]. In our study among 3–6-year-olds, children with high PEL had lower consumption of sugary foods and beverages and higher consumption of vegetables. An article describing eating styles derived from a principal component analyses among children in the DAGIS survey [39] claims that children with high PEL were less likely to have an eating style labeled as sweets-and-treats pattern. On the contrary, high vegetable consumption was characteristic of the vegetables-and-processed meats pattern, which was less often found among children with high PEL [39]. These dissimilarities in results, reflecting the vegetable consumption in different PEL groups among the same sample, stress the importance of applying several kinds of methods and viewpoints when examining eating behavior.

Our results indicating PEL differences in children’s food consumption differ somewhat when comparing the information derived from FFQs and food records. Measuring children’s VFB consumption frequency with FFQ, which provides information on food consumption outside preschool hours, revealed no PEL differences. On the contrary, the food record providing information on consumed amounts for one to three days indicated that children with low PEL ate less vegetables than children with high PEL. Food records do not provide valid information on the general diet, since foods eaten widely fluctuate daily [40]. In our data, for example, some children had not eaten any of the sugary foods and beverages or VFB focused on in our assessment during the three days of keeping the food record. Food records can, however, provide reliable and more detailed estimates of the mean intakes of frequently consumed foods. Regarding VFB, portion sizes probably differ despite similar frequency of consumption, thus yielding PEL differences in the actual amount eaten by the children.

We did not find PEL differences in children’s HCC, used as an indicator of children’s long-term stress. HCC is reportedly higher among preschoolers with low PEL in both Canada [41] and the Netherlands [42]. A Swedish study among one-year-olds, however, found no association between PEL and HCC, although higher HCC were found among children with mothers who were unemployed or on sick leave [43]. These kind of differences in results can reflect, for example, country-specific implications regarding how SES affects individuals and families. We noted, however, a large variation in children’s HCC. Large variation in HCC among young children and even higher HCC values than in our study have been reported previously by Karlen et al. [43]. HCC is generally higher among children than adults [44] and among children, extreme values have been associated with particular medication, such as hydrocortisone treatment in adrenal insufficiency [45]. In addition, associations exist between maternal stress during pregnancy and higher HCC among one- and three-year-old children but not among older children [43]. Unfortunately, we have no background data to explain the extreme values in our sample. As we only report PEL differences in the HCC and do not need to rule out alternative explanations of HCC, we decided not to exclude the extreme values, which could otherwise be treated as outliers.

We generally found fewer PEL differences in children’s EBRBs and long-term stress than expected. Despite this positive result, many children with low PEL may end up having unhealthier lifestyle when they grow older. This prediction relates to the presence of SES differences in many EBRBs among the adult population also in Finland [46,47]. One reason for the inability to demonstrate PEL differences in EBRBs and long-term stress in this study is the possibility of not having captured children with the lowest PEL (and SES). The study’s participating parents were quite highly educated, when compared to the educational level of the whole Finnish population [48]. If the lack of differences in EBRBs and long-term stress according to PEL, as demonstrated in our study, reflects the actual conditions among this age group, preschool could be an ideal context for preventive interventions. Preschool-based intervention could target both the interaction between personnel and children which associates with lower stress among children [49] and eating-related practices which in turn associate with children’s better quality diet [50]. In Finland, approximately 74% of the preschool age group attends preschool [51]; consequently, preschool is a suitable arena to reach children from different SES groups.

Another aspect relevant to examining SES differences is the choice of SES indicator. As shown in our DAGIS study, EBRB differences can be apparent or not, depending on whether the indicator of SES is based on maternal or paternal education [52]. Additionally, time spent in preschool possibly attenuates SES differences in children’s EBRBs and stress. Finnish preschools are guided by the Law of Early Childhood Education [53], implemented by the National Core Curriculum for Early Childhood Education and Care [54]. All municipal preschools, for example, are obliged to provide healthy food, which is free of charge and appropriate, and facilitate an appropriate amount of daily PA [54]. Consequently, future research could determine whether SES differences in EBRBs exist, by separately examining time spent at home and at preschool.

The main strength of the DAGIS survey is its exhaustive nature. We collected data related to several EBRBs and long-term stress among children by including multiple levels affecting a child. We also used different types of measurements, namely questionnaires, objective measures, and observation, to diversify our understanding of the path leading to SES differences in being overweight and obese. The survey was also conducted in different parts of Finland, covering participants from both rural and urban areas, and in municipalities with a diverse population based on their SES. Participants do not, however, represent the whole Finnish population.

Of those invited to participate in the study, only approximately one fourth participated. This low participation level may have distorted the results, making them more homogenous than is typical of the whole population. Due to the comprehensiveness of the survey, the burden for participating families was considerable, which probably lowered the willingness to participate. Since we have no information of the SES of the non-participants, we cannot rule out the possibility that participation and non-participation were SES-dependent. This is problematic for the generalizability of the results. Hence, future studies should place greater emphasis on the recruitment process to achieve higher participation rates. In the second phase of the DAGIS study, for example, we invested more in motivating preschool personnel, who were responsible for the recruitment of the parents.

The present paper presents the recruitment process of the first phase of the DAGIS study and the main PEL differences in children’s EBRBs and long-term stress. This leads to the second phase, a preschool-based and family-involving intervention, which aims to both enhance children’s EBRBs and stress regulation and diminish their observed SES differences. The intervention is built to be preventive in accordance with Proportionate Universalism [55], meaning it is adjusted to the needs of children with low SES. Therefore, the intervention targets both the predictors of EBRBs considered most important for the low SES group, and, as presented in this paper, the EBRBs in which the children with low PEL group scored lower.

## 5. Conclusions

There is limited evidence to suggest SES differences in EBRBs and long-term stress in this age-group, although some clear differences exist. The DAGIS intervention aims to diminish SES differences in preschool children’s screen time and consumption of sugary foods and VFB, that is, the EBRBs less favorable among children with low PEL. The DAGIS intervention is conducted at preschools and engages families as well. In preschool, pedagogical competence and practices related to PA and eating equally affect all children, regardless of their SES. Consequently, future studies could examine whether SES differences in children’s EBRBs and long-term stress are attenuated by their time spent at preschool.

## Figures and Tables

**Table 1:** Sample characteristics of the DAGIS study presented as percentage or mean, standard deviation (SD), and range.

Characteristics	*n*	Percentage	Mean (SD)	Range
**Parent**				
Parental status ^1^, mother	707	88		
Two-parent household	804	96		
Educational level	792			
Low (≤high school level education)	232	29		
Middle (Bachelor’s degree or equivalent)	327	40		
High (≥Master’s degree)	233	29		
**Child**				
Gender, girls	863	48		
Age, years	864		4.7 (0.9)	2.8–7.2
Overweight or obese ^2^	809	12		
Moderate to vigorous physical activity (minutes/hour)	773		5.6 (1.7)	2.0–11.9
Sedentary time (minutes/hour)	773		28 (4.0)	16–40
Screen time (minutes/day)	768		111 (48.5)	4–290
FFQ: Consumption frequency (times/day)				
Sugary everyday foods ^3^	806		0.9 (0.8)	0–8.4
Sugary treats ^4^	808		0.9 (0.5)	0–4.0
Sugary beverages ^5^	807		0.6 (0.7)	0–7.1
Vegetables ^6^	810		1.7 (1.0)	0–6.0
Fruit and berries ^7^	812		1.3 (0.9)	0–7.7
3-day food record: Consumption (g/day)				
Sugary everyday foods ^3^	813		105 (87)	0–552
Sugary treats ^4^	813		37 (29)	0–184
Sugary beverages ^5^	813		87 (100)	0–750
Vegetables ^6^	813		72 (49)	0–312
Fruit and berries ^7^	813		105 (81)	0–578
Hair cortisol concentration (pg/mg)	599		41 (77)	0.24–880

FFQ food frequency questionnaire; ^1^ Parental status of the guardian who filled in the survey questionnaire; ^2^ International body mass index (BMI) cut-off points for children [29] ^3^ Flavored yogurt and quark; puddings; sugar-sweetened cereals and muesli; berry, fruit and chocolate porridge with added sugar; and berry and fruit soups with added sugar; ^4^ Ice cream; chocolate; sweets; cakes, cupcakes, sweet rolls, Danish pastries, pies and other sweet pastries; and sweet biscuits and cereal bars; ^5^ Soft drinks; flavored and sweetened milk- and plant-based drinks; and sugar-sweetened juice drinks; ^6^ Fresh vegetables; and cooked and canned vegetables; ^7^ Fresh fruit; and fresh and frozen berries.

**Table 2:** Adjusted means and 95% confidence intervals (95% CI) ^1^ for EBRBs according to parental educational level (low/middle/high) from Ancovas (LSD, with Sidak corrections).

EBRBs	Parental Educational Level			
	Low Mean (95% CI)	Middle Mean (95% CI)	High Mean (95% CI)	*p*-Value
Sedentary time (minutes/hour) (*n* = 730)	28.2 (27.8, 28.7)	27.9 (27.6, 28.3)	28.2 (27.7, 28.6)	1.0 Low vs. High 0.71 Low vs. Middle 0.81 Middle vs. High
Moderate to vigorous physical activity (minutes/hour) (*n* = 726)	5.6 (5.4, 5.8)	5.5 (5.4, 5.7)	5.6 (5.4, 5.8)	1.0 Low vs. High 0.90 Low vs. Middle 0.94 Middle vs. High
Screen time at home (minutes/day) (*n* = 721)	118 (112, 124)	111 (106, 116)	104 (97, 110)	**0.01 Low vs. High** 0.26 Low vs. Middle 0.23 Middle vs. High
Total consumption frequency of sugary everyday foods ^2^, treats ^3^, and beverages ^4^ (times/day) (*n* = 709)	2.2 (2.0, 2.3)	2.1 (2.0, 2.2)	1.9 (1.8, 2.1)	0.07 Low vs. High 0.84 Low vs. Middle 0.23 Middle vs. High
Consumption frequency of sugary everyday foods (times/day) (*n* = 716)	0.8 (0.7, 0.9)	0.8 (0.7, 0.8)	0.7 (0.6, 0.7)	0.06 Low vs. High 0.91 Low vs. Middle 0.15 Middle vs. High
Consumption frequency of sugary beverages (times/day) (*n* = 713)	0.5 (0.4, 0.6)	0.4 (0.4, 0.5)	0.4 (0.3, 0.4)	**0.03 Low vs. High** 0.28 Low vs. Middle 0.55 Middle vs. High
Consumption frequency of sugary treats (times/day) (*n* = 712)	0.8 (0.7, 0.8)	0.8 (0.7, 0.8)	0.8 (0.7, 0.8)	1.0 Low vs. High 0.41 Low vs. Middle 0.85 Middle vs. High
Total consumption frequency of vegetables, fruit, and berries (times/day) (*n* = 718)	2.5 (2.4, 2.7)	2.6 (2.4, 2.7)	2.7 (2.5, 2.9)	0.60 Low vs. High 0.98 Low vs. Middle 0.76 Middle vs. High
Consumption frequency of vegetables ^5^ (times/day) (*n* = 724)	1.4 (1.3, 1.5)	1.4 (1.3, 1.5)	1.6 (1.5, 1.7)	0.17 Low vs. High 1.0 Low vs. Middle 0.15 Middle vs. High
Consumption frequency of fruit and berries ^6^ (times/day) (*n* = 721)	1.1 (1.0, 1.2)	1.1 (1.0, 1.2)	1.1 (1.0, 1.2)	0.99 Low vs. High 0.95 Low vs. Middle 1.0 Middle vs. High

EBRB, energy balance-related behavior; Statistically significant results at the level *p* < 0.5 are in bold; The analyses are adjusted for a respondent’s parental status (mother/father), a child’s gender (girl/boy) and age (continuous), and the research time (autumn/winter/spring). In addition, the analyses of Food frequency questionnaire (FFQ) variables measuring the consumption frequency of different foods and beverages are adjusted for time spent at preschool (continuous); ^1^ Geometric means and 95% CIs for LG10 transformed and back-transformed variables from FFQ; ^2^ Flavored yogurt and quark; puddings; sugar-sweetened cereals and muesli; berry, fruit and chocolate porridge with added sugar; and berry and fruit soups with added sugar; ^3^ Ice cream; chocolate; sweets; cakes, cupcakes, sweet rolls, Danish pastries, pies and other sweet pastries; and sweet biscuits and cereal bars; ^4^ Soft drinks; flavored and sweetened milk- and plant-based drinks; and sugar-sweetened juice drinks; ^5^ Fresh vegetables; and cooked and canned vegetables; ^6^ Fresh fruit; and fresh and frozen berries.

**Table 3:** Odds ratios (OR) and 95% confidence intervals (CI) for being in the highest quintile of consuming sugary everyday foods, sugary treats, sugary beverages, vegetables, and fruit and berries (3-day food record) or levels of long-term stress according to parental educational level (low/middle/high) from logistic regression analyses.

EBRBs and Long-Term Stress	Parental Educational Level	OR (95% CI)	*p*-Value
Consumption of sugary everyday foods ^1^ (g/MJ) (*n* = 766), highest quintile ≥ 31	Low	1.4 (0.9, 2.3)	0.17
	Middle	**1.7 (1.1, 2.6)**	**0.02**
	High	ref.	
Consumption of sugary treats ^2^ (g/MJ) (*n* = 766), highest quintile ≥ 10	Low	1.3 (0.8, 2.0)	0.31
	Middle	1.1 (0.7, 1.6)	0.82
	High	ref.	
Consumption of sugary beverages ^3^ (g/MJ) (*n* = 766), highest quintile ≥ 25	Low	1.3 (0.8, 2.1)	0.22
	Middle	1.0 (0.7, 1.6)	0.95
	High	ref.	
Consumption of vegetables ^4^ (g) (*n* = 765), highest quintile ≥ 109	Low	**0.6 (0.4, 0.96)**	**0.04**
	Middle	0.8 (0.5, 1.1)	0.18
	High	ref.	
Consumption of fruit and berries ^5^ (g) (*n* = 766), highest quintile ≥ 164	Low	0.8 (0.5, 1.3)	0.43
	Middle	1.1 (0.7, 1.6)	0.74
	High	ref.	
Hair cortisol concentration (pg/mg) (*n* = 559), highest quintile ≥ 56	Low	0.8 (0.5, 1.4)	0.41
	Middle	0.7 (0.4, 1.2)	0.22
	High	ref.	

Analyses are adjusted for a respondent’s parental status (mother/father), a child’s gender (girl/boy) and age (continuous), and the research time (autumn/winter/spring); Odds ratios that are statistically significant at level *p* < 0.05 are in bold; ^1^ Flavored yogurt and quark; puddings; sugar-sweetened cereals and muesli; berry, fruit and chocolate porridge with added sugar; and berry and fruit soups with added sugar; ^2^ Ice cream; chocolate; sweets; cakes, cupcakes, sweet rolls, Danish pastries, pies and other sweet pastries; and sweet biscuits and cereal bars; ^3^ Soft drinks; flavored and sweetened milk- and plant-based drinks; and sugar-sweetened juice drinks; ^4^ Fresh vegetables; and cooked and canned vegetables; ^5^ Fresh fruit; and fresh and frozen berries.

## Appendix A

**Table A1:** Data assessment methods and available data at different levels in the DAGIS survey 2015–2016.

Data Assessment Method	Measures	*n*	Additional Information
Child level			
Anthropometrics	Weight, height, waist circumference	810	Measured by research staff
Accelerometer	Physical activity, sedentary time, sleep	821	Actigraph worn 7 days by children
Food frequency questionnaire (FFQ)	Diet	819	Previous week, filled in by guardian
Home food record	Diet	813	1–3 days, filled in by guardian
Preschool food record	Diet	823	2 days, filled in by preschool personnel
Children’s Behavior Questionnaire	Self-regulation	751	Filled in by guardian
Saliva samples from a weekend day	Stress regulation	650	5 samples, collected by parents
Saliva samples from a preschool day	Stress regulation	650	2 samples, collected by preschool personnel
Hair sample	Long-term stress	599	Collected by preschool personnel
Sedentary behavior diary	Types of sedentary behavior (e.g., screen time), preschool time, wake up time, and bedtime	823	7 days, filled in by guardian
Home setting			
Guardians questionnaire in connection to consent	Gender of the child, own and spouse’s highest educational level	892	In paper form
Guardian’s questionnaire	Home physical and social environment, guardian’s gender and employment status, a child’s age, household income	809	In paper form or web-based questionnaire
FFQ of mother	Diet	802	Previous week, paper form
FFQ of father	Diet	660	Previous week, paper form
Preschool setting, group level			
Preschool personnel’s questionnaire	Socioeconomic status, Preschool’s physical, social, and policy environment	378	In paper form
Contact person’s extra questionnaire	Preschool’s practices related to EBRBs	146	In paper form
Observation	Preschool’s physical and social environment related to EBRBs	146	Conducted by research staff
Study week schedule of the preschool group	Physically active/sedentary activities to assist the interpretation of accelerometer data	133	In paper form, filled in by preschool personnel
Preschool setting			
Preschool principal’s questionnaire	Preschool physical, social, and policy environment	60	Web-based (http://w3.webropol.com/)
Preschool food service’s questionnaire	Kitchen type, practices concerning the serving of the food, serving frequency of different foods, opinions and attitudes	55	Paper form, filled in by food service personnel
Map Grid Database	In the area at 1 km and 5 km radius of the preschool: population’s structure, education, main type of activity and income, households’ stage in life and income	66	Register data from Statistics Finland https://www.stat.fi/tup/ruututietokanta/tietosisalto_en.html
General data			
Day of the field visit	Season of the data collection		

EBRB, energy balance-related behavior.
